# The Australasian Medical Congress

**Published:** 1920-10-23

**Authors:** 


### THE AUSTRALASIAN MEDICAL CONGRESS.

Immense interest was taken by all sorts and conditions of men in the Australasian Medical Congress held at Brisbane (Queensland) from August 23 to August 29, the first Congress since that held at Auckland (New Zealand) in 1911. About 300 medical men were present. Dr. W. Taylor (Brisbane) presided. Five of the sectional presidents were of Melbourne: Drs. R. R. Stowel, W. J. Penfold, J. H. L. Cumpston, H. C. Maudsley, and A. L. Kenny. Others were Drs. F. Barrington and E. H. Molesworth, of Sydney. The delegates included many physicians and surgeons who had seen service on the fields and in the hospitals of the Great War.

Dr. Taylor in his presidential address referred to the effects on the human system of new methods practised in the war—hypnotism being instanced, and he also advised his brethren to take strong concerted action in the matter of public health. This they could do by means of the British Medical Association, "which could do great work in prevention of disease by the influence which it might exert on Governments and local authorities which, with the best intentions, were proverbially slow to act unless some dire epidemic threatened. They did not fully realise that prevention was not only better than cure, but was much cheaper in the long run." Dr. Taylor also referred to the nationalisation of medicine, which so many medical men seem to desire. Its effect, he thought, would depend upon the conditions under which medical men would be required to work. "If the newly qualified practitioner began with £500 yearly, received automatic increases each two or three years, and worked only eight hours daily, the profession would attract men of ability."

Admirable addresses were delivered by Col. Dr. R. J. Millard concerning naval and military work at the Front and preventive methods in Australia, and by Dr. Herbert Marks (Sydney), president of the section for otology, rhinology, and laringology.

The address on "Physiological Factors in the Development of an Australian Race," by Professor Osborne, professor of physiology at the University of Melbourne, had been keenly anticipated, and was heard by a crowded audience in the (Brisbane) Albert Hall.

Professor Osborne began with the influence of climate on racial characteristic. "We are a white population and intend to remain white," he remarked. The influence of food upon racial development was next dealt with, and, turning to disease, Professor Osborne explained that "All Great Britain's attempts to colonise the tropics have failed. This may be attributed to disease, and not to climate. We in Australia enjoy singular immunity from tropical disease, partly owing to the absence of a large native population, which is always a reservoir of infection. . . . It has been noted that disease is generally milder in Australia than in the countries from which it comes. . . . If disease is to be kept under, cleanliness is essential. If people would keep clean there would be no hookworm or typhoid, and little tuberculosis or influenza. It is a sad reflection that man's greatest enemies—disease, war, narcotism, superstition, and tyranny—are mostly of his own creation. Australia can be as free from disease as it is possible for a country to be, and here is a grand opportunity for statesmen to found a national policy of health." In conclusion, referring to the influence of climate on race development, Professor Osborne said that scientists have not yet sufficient data to enable them to form a proper opinion as to the possibility of white races settling in the tropics. Those who touch on the subject must be careful, for great issues depend upon the conclusions reached. Climate and disease are so closely connected that not one instance of climate acting alone can be given. The climate of the temperate regions of Australia is conducive to the growth of a robust population, but Australia must first have (1) National administration of preventive medicine, including the treatment of people after school life; (2) a form of national medical service that would not rob the practitioner of responsibility and initiative; (3) the development of welfare work in all business houses; (4) more flexibility in habits and greater readiness to learn from other people—the adobe houses of Mexico, for instance, would be healthier than the corrugated-iron shack, and the siesta has never had a trial; (5) no matter what salutary machinery is evolved, there must ever remain the personal choice between indulgence and restraint, between folly and wisdom, between virtue and vice.

Hopes of progress depend mainly on education in its widest sense—musical and artistic sides included—and particularly on the extension of secondary education. It would be of little service to the world if Australians became merely a nation of athletes.

On Sunday morning, August 29, the Australasian Medical Congress met in full session to deal with resolutions forwarded by the various sections, and also for the purpose of coming to a decision as to future control administration of Congresses.

It was resolved that the Australasian Medical Congress should cease to exist as such; its affairs to be wound up by the present executive. Arising out of a discussion at the last Congress (1911), a resolution was read from the Federal Committee of the B.M.A. in Australia to the effect that the Committee would be prepared to carry on the B.M.A. Medical Congress, in the event of the Australasian Medical Congress being wound up, on behalf of the B.M.A. in Australia, in collaboration with the New Zealand branches.

An invitation was received from Hobart for the next Congress. This was handed over to the B.M.A.

